# Olfactory neuroblastoma: diagnosis, management, and current treatment options

**DOI:** 10.3389/fonc.2023.1242453

**Published:** 2023-10-16

**Authors:** Alicia Tosoni, Vincenzo Di Nunno, Lidia Gatto, Giacomo Corradi, Stefania Bartolini, Lucia Ranieri, Enrico Franceschi

**Affiliations:** ^1^ Nervous System Medical Oncology Department, IRCCS Istituto delle Scienze Neurologiche di Bologna, Bologna, Italy; ^2^ Department of Oncology, Azienda Unità Sanitaria Locale (AUSL) Bologna, Bologna, Italy; ^3^ Department of Medical and Surgical Sciences, University of Bologna, Bologna, Italy

**Keywords:** esthesioneuroblastoma, olfactory neuroblastoma, chemotherapy, radiotherapy, surgery

## Abstract

Olfactory neuroblastoma (ONB) is a rare neoplasm originating from the olfactory neuroepithelium representing 3-6% of tumors of the sinonasal tract. ONB require multi-disciplinary care. Historically, the gold standard surgical procedure for ONB has been open craniofacial resection. In the last years, endoscopic endonasal approaches have been largely introduced with lower complication rates, shorter hospital stay, and similar clinical outcome. Radiotherapy plays an important role in the management of ONB, however there are not generally accepted recommendations for its application. Although there is agreement that multimodal therapy is needed, the optimal use of chemotherapy is still unknown. The rarity of the disease, makes difficult to draw definitive conclusions about the role of systemic treatment in induction and concomitant setting.

## Epidemilogy

Olfactory neuroblastoma (ONB), also called esthesioneuroblastoma, is a rare neoplasm originating from the olfactory neuroepithelium with neuroblastic differentiation, representing 3-6% of tumors of the sinonasal tract. Since its initial description in 1924, more than 1000 cases of ONB have been described worldwide (1).

It most often presents in the superior nasal cavity including the lamina cribrosa of the ethmoid bone and the superior nasal concha. It is a locally aggressive neoplasm that may involve local structures such as the skull base and orbits, and has a tendency to metastasize in 20-48% of cases. The typical sites of metastasis are cervical lymph nodes (10-33% of patients), bones, and lungs (2). ONB demonstrates an unimodal distribution with a more common presentation in adulthood around the age of 50-60 years (3).

In a retrospective surveillance, epidemiology, and end results (SEER) registry analysis 636 patients were identified in the period 1977-2016, the majority being male (59.7%), and Caucasian with a median age of 51.4 years. The highest incidence of disease onset occurred in patients between the ages of 18‐39 years (17.5%) and 40‐59 years at diagnosis (46.1%) and the majority of patients were diagnosed with a primary tumor involving the nasal cavity (78.3%) (4). Interestingly, another analysis on SEER data indicates that patients of the lowest socioeconomic status (SES) were almost 85% more likely to present with advanced-stage cancer than patients in the highest SES. Notably the same study reported that patients with lower SES exhibited higher mortality and a dramatic 70% worse disease specific survival (DSS) compared with the highest SES (5).

## Diagnosis

**Figure 1:**
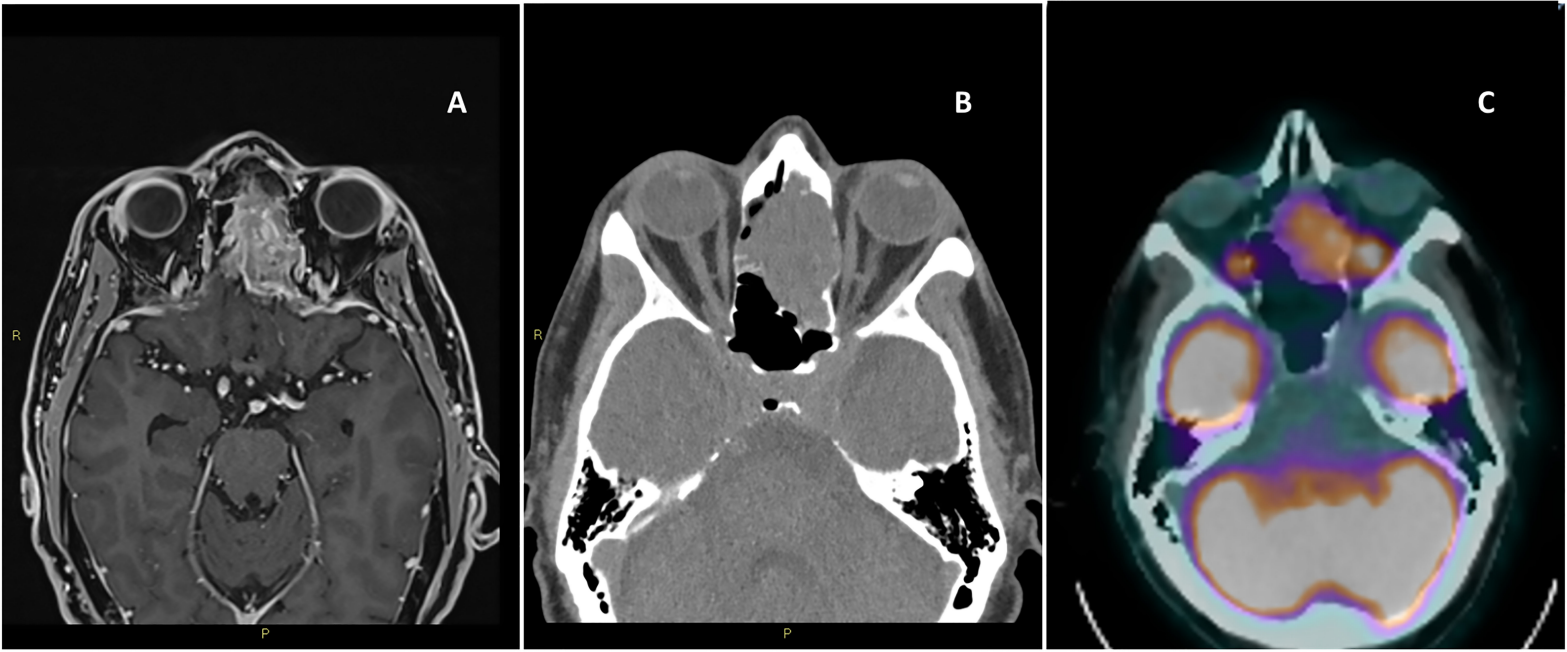
Magnetic resonance imaging of the brain T1 with contrast, axial view **(A)**, computer tomography of the brain axial view without contrast **(B)**, positron emission tomography axial view **(C)**.

Nasal obstruction followed by epistaxis are typical early manifestations (6, 7). Hyposmia and anosmia can precede the diagnosis of ONB by several years (4). Other symptoms are related to the anatomic structures affected by the local invasion. Visual or ocular disorders could be related to the extension into the orbit. Intracranial invasion can produce headache, and manifestations of inappropriate antidiuretic hormone secretion (SIADH). Because of the aspecific nature of early symptoms, delayed diagnosis is frequent with an median time of 6–12 months between symptom onset and diagnosis (8). A “dumbbell-shaped” mass extending across the cribriform plate is one of the most characteristic radiological findings of this tumor (9). Computer tomography (CT) and/or magnetic resonance imaging (MRI) of skull base, paranasal sinuses, and neck are needed for qualitative evaluation and staging. CT is an helpful initial study, and can better describe the bone involvement, whereas MRI better evaluate the orbital and intracranial infiltration On MRI ONB is most typically hypointense on T1 and could appear as a contrast enhancing lesion. T2 shows and isointense or hyperintense mass (10). Full body CT and positron emission tomography scans are indicated in the diagnostic work up to determine the systemic extent of disease (11). (**Figure 1**) Some reports suggest that Gallium68-DOTATOC PET could be additionally used to assess the somatostatin receptor expression, demonstrating an utility in the diagnosis, staging, and treatment-response monitoring of patients with ONB (12).

Biopsy is mandatory for diagnosis, and it is generally performed after imaging. The great variety of different histotypes occurring primarily in the sinonasal tract together with the presence of limited biopsy material, pose significant diagnostic difficulties for the pathologist requiring specific knowledge and availability of immunohistochemical and molecular techniques. In recent years, the increasingly frequent participation in work groups has favored the development of a pathologists network with specific skills in sinonasal region area as well (13). For correct diagnostic identification, several biomarkers have been identified, including: synaptophysin, chromogranin, S-100, CD-56 and neuron-specific haemolysis (NSE). These biomarkers appear to be of fundamental importance for diagnosis, but have not yet been included among the prognostic factors. Proliferation marker studies using Ki-67 reveal a high proliferative index of 10–50%. Studies are increasingly focusing on the molecular profile, even for these extremely rare diseases (14).

A disease specific grading system for ONB has been described by Hyams in 1988, in which the disease is stratified into four grades ranging from most differentiated (grade I) to least differentiated (grade IV) on the basis of mitotic activity, nuclear polymorphism, amount of fibrillary matrix, rosette formation, and amount of necrosis (**Table 1**). Recent evidence suggests a correlation between the Hyams grading and clinical outcome, with high-grade (grade III/IV) tumors associated with worse survival outcomes as compared with low-grade (grade I/II) tumors (5-year survival rate of 80% and 54% and a 10-year survival rate of 67% and 36% for low and high grade, respectively) (15). Similarly, the meta-analysis conducted by Dulguerov et al. confirmed that Hyams grading was significant associated with survival, showing a 5-years survival rate of 56% and 25% for low grade and high grade respectively (16). On the other hand, it is important to note that Hyams system is a subjective scale, leading to variable grading between pathologists. Furthermore, biopsy may also lead to sampling error when the entire tumor is not examined, as different parts of the tumor may contain different Hyams grades.

**Table 1:** Hyams grade for olfactory neuroblastoma.

	Grade I	Grade II	Grade III	Grade IV
Architecture	Lobular	Lobular	Variable	Variable
Fibrillary matrix	Prominent	Present	Minimal	Absent
Mitosis	Absent	Present	Prominent	Marked
Necrosis	Absent	Absent	May present	Common
Nuclear pleomorphism	Absent	Moderate	Prominent	Marked
Rosettes	Homer Wright	Homer Wright	Flexner- Wintersteiner	Flexner- Wintersteiner

However, based on published data, Hyams grading may represent an important factor for decision making in therapeutic strategies such as induction chemotherapy and adjuvant radiotherapy (RT), and may also be considered in surveillance protocols.

## Staging

Different staging systems have been proposed. The most commonly applied was proposed by Kadish and colleagues in 1976. This staging system classifies three categories: stage A, tumor restricted to the nasal cavity; stage B, tumor extending to the paranasal sinuses; stage C, tumor extending beyond the paranasal cavities. The Kadish classification was later modified by Morita et al. (17) designating the class D that includes patients with cervical lymph node metastases (18). A significant differences in clinical outcome has been showed between the four groups of the modified Kadish classification, in particular the overall survival (OS) and DSS rates at 10 years to be 83.4% and 90%, respectively, for patients with stage A disease; 49% and 68.3% for patients with stage B disease; 38.6% and 66.7% for patients with stage C disease; and 13.3% and 35.6% for patients with stage D disease (19).

Other proposed staging systems include the tumor-nodal-metastasis (TNM) system by the American Joint Committee on Cancer, and a modified TNM version by Dulguerov (20).

The Dulguerov system, uses the TNM classification including the imaging data, it separates patients with or without sphenoid sinus disease, as well as differentiates between those intracranial but extradural tumors from those with true brain involvement. (**Table 2**).

**Table 2:** Staging systems of olfactory neuroblastoma.

Kadish staging	
A Confined to nasal cavity
B Involves nasal cavity and paranasal sinuses
C Extends beyond the nasal cavity and paranasal sinuses
Morita modification	
A Confined to nasal cavity
B Involves nasal cavity and paranasal sinuses
C Extends beyond the nasal cavity and paranasal sinuses
D Regional or distant metastasis
Dulguerov Modified TNM Staging	
Primary tumor	
T1 Nasal cavity/paranasal sinuses (not sphenoid or superior most ethmoid)
T2 Includes sphenoid with extension to/erosion of cribriform plate
T3 Extends into orbit or anterior cranial fossa without dural invasion
T4 Tumor involving brain
Lymph nodes	
N0 No cervical limph node metastasis
N1 Any cervical lymph node metastasis
Distant metastasis	
M0 No metastasis
M1 Distant metastasis

## Therapy

The complex anatomy of this region with proximity to vital structures such as the orbit, skull base, and brain, makes complete surgical resection with sufficient margins not often feasible.

This tumor location require multidisciplinary care that includes medical oncologists, neurosurgeons, head and neck surgeons, pathologists and radiation oncologists. Local treatment with surgery is frequently recommended for primary therapy. While a combination of surgery and postoperative RT is indicated for the management of more local advanced resectable cancers. Locally advanced disease often requires a multidisciplinary approach with surgery, radiation, and systemic therapy serving as key components of treatment (21).

## Surgery

Surgery remains a fundamental step in the therapeutic process. The extent of resection (R0 and R1) has been shown to be an independent factor for overall survival and event free survival (22).

Historically, the gold standard surgical procedure for ONB has been open craniofacial resection or transfacial surgery. In the last two decades, endoscopic endonasal approaches have been introduced with lower complication rates and shorter hospital stay (23). In 2019, the International Consensus Statement on Endoscopic Skull Base Surgery suggested that Kadish stage A and B tumors should be treated endoscopically. Kadish C tumors should be performed endoscopically only if negative margins can be obtained, while Kadish C tumors involving the orbit, spread lateral to the orbital axis, hard/soft palate, or midface should be treated with an open surgery (24).

Multiple factors may be considered in choosing the optimal surgical approach, including tumor size and location, patient comorbidities, and experience of the surgical team. Notably, when selecting the optimal surgical approach, the surgeon must consider the approach that will allow for a negative margin resection and adequate reconstruction.

In patient with intracranial involvement, when anatomic barriers preclude the surgeon from gross total resection, a combined intracranial and extracranial approach could be required, and in these patients an endoscopic surgical technique may be combined with a transcranial neurosurgical approach. However, the continue evolution of endoscopic technique, that allows the visualization of the suprasellar region in a similar fashion to that of bilateral subfrontal approach, makes this combined approach less used (2).

It is difficult to compare the clinical outcome between endoscopic and open surgical approach, due to the rarity of this tumor that limits evaluation of large‐scale studies. Several reviews and meta‐analyses comparing outcomes between endoscopic and open surgery have shown at least equivalent survival data. A systematic review and pooled‐data analysis of 226 patients demonstrated that there was no difference in survival outcomes between endoscopic and traditional open surgery for T1 and T2 sinonasal malignancies (24).

Schwart et al. (25) compared the results of endoscopic surgery (ES) with transcranial surgery (TS) for ONB over two different time periods (before 2012 and 2012-2017) to assess the evolution of results over time. In particular, before 2012, the meta-analysis showed that ES was already advantageous compared to the other surgical approaches: gross total resection (GTR) 98.1% versus 85.2% and progression free survival (PFS) 8% versus 22.1%. Major complications included meningitis, CSF leaks and infections. In particular CSF leak is one of the more important complications, being reported in 6% of patients after TS, 7.2% after ES, and 18% in combined cranionasal approach (26). However, the use of repair strategies, such as the pedicled nasoseptal flap procedure, appear to be effective, being post operative CSF leak repair failure reported in only 5.3% of patients (27).

In subsequent years, TC approach continues to be accompanied by a relatively high rate of complications of 52.9%, and purely ES continue to proliferate, demonstrating high 5-year overall survival OS (82–97%).

Studies of ES versus TS approaches for pooled groups of sinonasal malignancies, including ONB, have shown comparable GTR rates between the two (23, 24).

Spielman DB et al., reported on 339 ONB patients undergoing ES for different stages and grades of disease. Negative margins have been achieved in 86.9% of cases with an overall recurrence rate of 10.3% and 5‐year survival of 91.1% (28). On the other hand, Patel et al. reported on 151 patients from 17 institutions who underwent TS, of these 77% of tumors with Kadish stage C. Overall 60% had received treatment before TS, radiation therapy or chemotherapy. Postoperative adjuvant RT and adjuvant chemotherapy were used in 60 patients. Treatment complications occurred in about 32% of patients with an OS of 78% and a recurrence free survival of 64% at 5 years (29).

The current practice appears to favor ES or combined approaches for early-stage, endoscopically accessible disease. For later-stage, more invasive disease, some can be resected successfully endoscopically, but TS could still be considered to achieve a maximal safe resection. Surgical management of the cervical lymph nodes for patients with ONB remains matter of debate. The incidence of cervical metastases at diagnosis is 5-8%, but the incidence of a later development increase to 20-25%. Despite this relative high incidence, surgical management of the neck is reserved for patients presenting with clinical or radiological evidence of neck disease (30).

## Radiotherapy

RT plays an important role in the management of ONB, however there are not generally accepted recommendations for its application. Different radiation approaches have been evaluated over the years, ranging from elective RT to treat Kadish stage A and B to pre/postoperative RT plus concomitant chemotherapy (31). Concomitantly, there has been an improvement in RT techniques over the years, reducing treatment-related toxicity and allowing the preservation of nearby vital structures. In patients with early stage disease (Kadish A and B) some studies (17, 32, 33) reported no survival differences between primary RT and combination treatment with surgery plus pre or post operative RT. However, in general an increase in tumor control has been reported when surgery is combined with RT, even if no consensus exist for the timing of the RT that can be used pre or postoperatively (32, 33). With regard to postoperative RT (PORT), a retrospective study based on SEER database confirmed no impact in OS in Kadish stage A and B, whereas a significant better OS was demonstrated in patients with more advanced stages (C and D), with an OS at 5 and 10 years of 70.7% and 53.4% with PORT versus 42.6% and 29.5% without PORT (34). It is generally accepted that higher stage lesions require the combination of surgery and RT (17, 31, 32) even if some studies (35, 36) suggest a combined approach for all stages. Considering tumor grade, low grade tumors can be treated with surgery alone if there are free tumor resection margins. Whereas RT is recommended for high grade tumors and low-grade tumors borderline resected, or residual or recurrent tumors (17, 31). In general, preoperative dose of 45 Gy and postoperatively dose of 50–60 Gy are indicated. For definitive RT, doses of 60–70 Gy should be recommended (31). Some retrospective studies analyzing the role of elective neck irradiation in patients with clinical N0 reported a significant reduced risk of cervical nodal regional recurrence, but this did not translate to a survival benefit.^33,34^ The safety of intensity-modulated RT (IMRT) in the management of ONB has been evaluated retrospectively over 3 years showing the absence of acute high-grade toxicity and infrequent cases of late toxicity, including: dysosmia (3.8%), hearing loss (3.8%), brain damage (1.9%), and temporal lobe necrosis (1.9%). No late ocular toxicity was observed (37). New radiation techniques such as particle-beam radiation therapy (PBRT), typically using accelerated proton or carbon-ion, has the advantage of a dose-focusing Bragg peak, which allows the radiation to penetrate in to the depth of the target and then terminate, sparing normal tissues beyond the target from unnecessary radiation (38, 39). Furthermore, carbon-ion beam is characterized by a higher linear energy transfer and a relative biological effectiveness which enables more effective cell killing through inducing more DNA double-bond damage. Because of their rarity, no standard of care in PBRT has been established for ONB. Preliminary data on retrospective series reported that this type of approach is well tolerated and that it is acceptable in terms of OS and PFS, in the absence of acute or late toxicities greater than or equal to grade 3 (40).

## Induction chemotherapy

The main goals of induction chemotherapy (IC) could be to allow in responding patients an organ preservation of critical structures like eye or brain, and to reduce the risk of distant metastasis (41). Furthermore an important advantage associated to IC is its potential role in predicting clinical outcome (42).

However, whether these goals are achieved has not been well established due to the rarity of the disease, heterogeneity of considered series, and lack of prospective studies. Data examining the utility of IC are limited to small series, reporting on Kadish C patients treated with various schemes and demonstrating a response rate (RR) of 25-100% (**Table 3**). The outcome reported by these series, suggests that Kadish C patients treated with a multimodality strategy including IC followed by surgery and RT could achieve similar survival of patients presenting with locally advance disease (OS at five years of 72%) (39, 40).

**Table 3:** Neoadjuvant treatment.

Author	Number of pts	Regimen	RR
Patil (41)	12	DDP+VP-16	66.7%
Fitzek (43)	9	DDP+VP-16	60%
Zappia (44)	2	DDP+VP-16	100%
Chao (45)	8	DDP+VP-16 or VCR+EX+doxo	25%
Wade (46)	8	VCR+EX	62%
Kim (47)	11	Ifo+VP-16+DDP	82%
Bartel (48)	4	Ifo+VP-16+DDP	75%
Modesto (49)	23	HDCT or DDP+VP-16 or CBCDA+5FU	74%

DDP cisplatin, VP-16 vepesid, VCR vincristin, EX endoxan, Doxo doxorubicin, ifo ifosfamide, CBCDA carboplatin, 5FU 5 fluorouracil, HDCT, high dose chemotherapy,

## Concomitant chemoradiotherapy

Local recurrence remains the major issue in the management of ONB (49). With the aim to increase local control some studies and referral centers advocate the use of concurrent chemoradiation (CT-RT) with cisplatin after surgery for patients at high risk of local recurrence (30). In a recent retrospective study (50), on 931 ONB patients who received CT-RT, a greater benefit has been reported (HR 0.22, P <.01) in comparison to patients treated with RT alone. Similarly, Sun et al. (51) reported results on 138 patients with non-metastatic ONB demonstrating that surgery followed by CT-RT achieved the best prognosis compared to patients treated with surgery alone and surgery plus RT. Xiong et al. (52) compared the prognosis of patients with different treatment modalities demonstrating that surgery followed by CT-RT yielded the best survival results. On the contrary, in a retrospective study in which 797 ONB patients were considered, it was found by multivariate analysis that the use of chemotherapy in addition to RT or surgery was associated with a reduced DSS (HR 2.78) and OS (HR 2.17) (53).

## Adjuvant chemotherapy

The efficacy of adjuvant chemotherapy to increase OS has been explored in some retrospective series. Miller et al. (50) compared survival among patients treated with surgery followed by RT alone to patients who underwent the same treatment followed by adjuvant chemotherapy, showing no increase in OS or recurrence free survival with the addition of adjuvant chemotherapy. In the Mayo Clinic retrospective review (54) adjuvant chemotherapy for patients with high grade, Stage C ONB was of benefit following complete resection leading to an increase in median time to relapse (35 and 10.5 months), and in OS (83 and 78 months respectively).

## Treatment of advance disease

Metastatic disease could develop in 12% of ONB patients with a median time of 15 months (55). Clinical reports suggest that ONB can be considered a chemosensitive tumor (42). However, due to the rarity of the disease, no standard chemotherapy regimen exists. One of the earliest studies on chemotherapy in the palliative setting has been published by Mayo Clinic (56) in which 10 patients with advanced disease were observed retrospectively after first-line treatment with platinum-based chemotherapy. The study reported chemotherapy response only in two of the four patients with high-grade tumors. OS was 44.5 months and 26.5 months in patients with low and high-grade tumors respectively. The study concluded that Hyams grade was an important predictor of treatment response, but was also related to a worse outcome. Marinelli et al. (55) conducted a systematic review and meta-analysis on 118 patients metastatic ONB treated in 48 studies demonstrating that the combination of chemotherapy with surgery and/or RT exhibited the best overall survival when compared to a single treatment modality. Platinum plus etoposide chemotherapy seems to be the most used regimen, even if not provide a survival benefit when compared with all other regimens.

## Treatment of recurrent disease

Recurrence has been showed in 30-60% of patients successfully (1-5 Garret) treated for the primary tumor. Recurrence tends to appear commonly after 5 years or more after initial treatment. Recurrence seems to develop before in high grade compared to low grade tumors (3.75 vs 5.7 years) (57). Patients more commonly developed a local recurrence (sinonasal 22.2%, intracranial 31.1%, cervical lymph nodes 33.3%), while metastatic recurrence has been demonstrated in only 13.3% of patients (57). No standard treatment exists for recurrent disease. Multidisciplinary discussion is needed to consider single or multimodality treatment that could comprise salvage surgery, targeted radiotherapy and/or chemotherapy based on recurrence location and previous treatments. Ni et al. (57) recent published on 64 recurrent ONB patients treated at Mayo Clinic, reporting the choice of salvage surgery in 69% (neck dissection in 51%), radiotherapy in 56%, gamma knife surgery in 20%, and chemotherapy in 26% of recurrent patients (Ni). In terms of chemotherapy, 70% of patients were treated with platinum based chemotherapy, 40% with taxanes, 50% with topoisomerase inhibitors and 30% with alkylating agents. The role of stereotactic radiosurgery to treat focal intracranial recurrence of ONB has been also evaluated in 27 recurrent patients unfit for open or endoscopic surgery, reporting a local control in 89% of tumors after a median of 36 months, without treatment complications (58). Salvage treatment seems to be effective with 63% of patients alive 5 years after recurrence. However subsequent recurrence has been reported in 20% of patients, frequently requiring additional therapy (57).

## Target treatments

Identifying potentially targetable genomic alterations in rare tumors is particular intriguing because no standard of care exists, and treatment is often extrapolated. The development and improvement of new sequencing technology, next-generation sequencing (NGS) has been applied and increasingly used to identify novel and rare cancer mutations, providing a molecular rationale for appropriate targeted therapy (**Table 4**). A comprehensive genomic profiling (59) was performed on 41 consecutive clinical cases of ONB using NGS to identify genomic alterations that could identify potential targeted therapies. 68% of ONB harbored genetic alterations, and approximately half featured at least one genetic alteration of therapeutic relevance. The most commonly altered gene was *TP53* (17%), with genetic alterations in *PIK3CA*, *NF1*, *CDKN2A*, and *CDKN2C* occurring in 7% of samples. In this interesting analysis data on individualized target treatment have been reported: one case of disease stabilization to everolimus for a tumor with a PIK3R2 mutation; two responses to sunitinib; and one stable disease in response to pazopanib and docetaxel. Topcagic et al. (60) explored a wide range of potentially targetable biomarkers in ONB samples using multiple molecular profiling platforms including NGS. The results showed mutations in *TP53*, *CTNNB1*, *EGFR*, *APC*, *cKIT*, *cMET*, *PDGFRA*, *DCH1*, *FH* and *SMAD4* genes. Multiple genes within the Wnt/β-catenin signaling pathway including *CTNNB1*, *APC* and *CDH1* exhibited mutations within this cohort. Multiple alterations in markers such as *ERCC1, TOPO1, TUBB3* and *MRP1*, which are known to reflect sensitivity to cisplatin, irinotecan, vincristine and combination therapy, have been identified. In one study, ONB was found to have in 28% of tumors, an amplifications of the targetable receptor tyrosine kinase *FGFR3* which could be a possible therapeutic target (61). Interestingly, Gallia et al. (62) showed a high frequency of deletions in the dystrophin (*DMD*) gene (86% of tumors) This high prevalence implicates an unexpected functional role for genes causing hereditary muscular dystrophies in ONB. The authors point to previous studies, which demonstrated the tumour-suppressive role of *DMD*, highlighting the potential utility of this specific aberration as a therapeutic target. (21) Recent case reports have shown a prolonged disease stabilization after treatment with sunitinib (63), and a partial response after treatment with the combination of sunitinb and cetuximab in one patient whose tumor harboring gene mutations in the genes encoding *EGFR, FGFR2, KDR*, and *RET (* 64). Case studies have reported disease stabilization in response to treatment with everolimus (65), and imatinib (61). Dunbar et al. reported on a metastatic ONB patient achieving a stable disease for 22 months with the antiangiogenic agent bevacizumab (66). A single case of a metastatic ONB, showing at the NGS a pathogenic fumarate hydratase mutation, achieved a prolonged partial response with pazopanib for over 4 years (67). Lastly, conflicting data have been reported on PD-L1 expression in ONB, with 0-40% of PD-L1 expression in tumor cells (60, 68), suggesting that there should be further investigation into the role of immunotherapy in ONB. Unfortunately, to systematically study the efficacy of targeting these individual pathways in rare cancers like ONB would be nearly impossible. Hence, novel clinical trial designs, such as basket trials, will be required to assess these approaches. As we know unequivocally that surgical resection currently comprises the cornerstone of ONB management, a “Window of Opportunity” trial to apply these agents prior to surgery could offer a possible avenue to test this strategy.

**Table 4:** Case reports on target therapies in recurrent olfactory neuroblastoma.

Molecular target	Treatment	Outcome
*PTCH1* splice site 395-1G>A (59)	Vismodegib	PFS 3 months
	Sunitinib	SD for 24 months
*PIK3R2* G87fs*14 (59)	Everolimus	SD for 12 months
*CTNNB1* T41I, *PTEN* splice site 210-2A>C, *ARID1A* Q1424*, *KDM5C* E375* (59)	Everolimus	PFS 3 months
	Pazopanib/docetaxel	SD for 24 months
*TP53* Loss, *KIT* amplification, *AXL-ARHGEF* fusion (59)	Sunitinib	PFS > 3 months
IHC+ *PDGFR-b* in stromal and endothelial cells (63)	Sunitinib	SD for 15 months
*EGFR* Mutation p.Arg521Lys exon13 (64) *KDR* Mutations p.Gln472His (exon11) and p.Val297lle (exon7) *FGFR2* Mutation p.Met186Thr exon5 *RET* Mutation p.Met1009Thr exon 18.	Cetuximab plus sunitinib	CR after 1 months
Not identified (65)	Everolimus plus cisplatin	SD > 24 months
Not identified (66)	Bevacizumab	SD for 22 months
*Fumarate Hydratase* Mutation Exon 10 K477dup (67)	Pazopanib	PR > 48 months

IHC+ Immunohistochemistry positivity, PFS progression free survival, SD stable disease, CR complete response, PR partial response. fs* frameshift mutation.

## The role of multimodality therapy

Although the majority of patients initially present with locally advance disease, the overall prognosis is high compared to other sinonasal tumors, with a 5-year overall and progression free survival estimated at 63% and 57% respectively. Nodal involvement at diagnosis, present in 21% of ONB patients, remains the major prognostic factor (49). Surgery alone has been considered as an adequate treatment only for small, low-grade tumors confined to the ethmoids when negative surgical margins can be obtain. For more advanced disease, or high grade disease a consensus has been obtained in the use of multimodality therapy including a complete surgical resection in combination with RT and/or chemotherapy (6, 36). IC could be considered for stage C tumors as a consequence of ONB chemosensivity to maximize the chance of optimal surgical resection or definitive RT, especially for high grade tumors who are known to have worse prognosis and higher chemosensivity. After surgical resection adjuvant RT must be considered in case of high grade and/or advanced stage tumors, or in presence of no clear or borderline margins. According to data from other head and neck tumors, cisplatin concurrent chemotherapy could increase radiation efficacy and decrease disease dissemination particularly in case of positive margins and nodal extension at diagnosis. RT alone or CT-RT is a possible approach for patients with low grade unresectable tumors, which could lead in case of response to a subsequent surgical approach.

## Conclusions

Clinical management of these rare disease has been improved in recent years. The progressive introduction of endoscopic surgery approaches has reduced patients perioperative morbidity, and seems to give, in high volume specialized centers, similar clinical outcome in comparison to open craniofacial resection. Another challenge in endoscopic approach in patients with intracranial disease has been the improvement in skull base reconstruction techniques, that allows combined surgical approaches also in locally advance disease. Evident improvements have been demonstrated in RT techniques. The introduction of particle-beam radiation therapy is ideally suited for dose escalation in complex anatomical sites, reducing toxicity of nearby critical tissues.

On the other hand, even if an agreement that multimodal therapy is needed (69), the optimal use of chemotherapy is still unknown. Clearly, the heterogeneity and rarity of the disease, makes difficult to draw definitive conclusions about the role of systemic treatment in induction setting, and its possible role in organ preservation. Likewise limited data are available about the use of concomitant CRT. Advances in molecular profiling could lead to the identification of new target therapies with new future therapeutic scenario.

## Author contributions

AT, GC wrote sections of the manuscript. All authors contributed to manuscript revision, read, and approved the submitted version. The publication of this article was supported by the "Ricerca Corrente" funding from the Italian Ministry of Health.
